# Deep Learning-Based Multinational Banknote Type and Fitness Classification with the Combined Images by Visible-Light Reflection and Infrared-Light Transmission Image Sensors

**DOI:** 10.3390/s19040792

**Published:** 2019-02-15

**Authors:** Tuyen Danh Pham, Dat Tien Nguyen, Chanhum Park, Kang Ryoung Park

**Affiliations:** Division of Electronics and Electrical Engineering, Dongguk University, 30 Pildong-ro 1-gil, Jung-gu, Seoul 100-715, Korea; phamdanhtuyen@dongguk.edu (T.D.P.); nguyentiendat@dongguk.edu (D.T.N.); pipetsupport@naver.com (C.P.)

**Keywords:** multinational banknote type and fitness classification, fitness value estimation, visible-light reflection image, infrared-light transmission image, deep learning

## Abstract

Automatic sorting of banknotes in payment facilities, such as automated payment machines or vending machines, consists of many tasks such as recognition of banknote type, classification of fitness for recirculation, and counterfeit detection. Previous studies addressing these problems have mostly reported separately on each of these classification tasks and for a specific type of currency only. In other words, there has been little research conducted considering a combination of these multiple tasks, such as classification of banknote denomination and fitness of banknotes, as well as considering a multinational currency condition of the method. To overcome this issue, we propose a multinational banknote type and fitness classification method that both recognizes the denomination and input direction of banknotes and determines whether the banknote is suitable for reuse or should be replaced by a new one. We also propose a method for estimating the fitness value of banknotes and the consistency of the estimation results among input trials of a banknote. Our method is based on a combination of infrared-light transmission and visible-light reflection images of the input banknote and uses deep-learning techniques with a convolutional neural network. The experimental results on a dataset composed of Indian rupee (INR), Korean won (KRW), and United States dollar (USD) banknote images with mixture of two and three fitness levels showed that the proposed method gives good performance in the combination condition of currency types and classification tasks.

## 1. Introduction

Recently, despite the growth of electronic financial transactions that have caused a decrease in the use of physical currency, transactions involving banknotes are still playing an important role in daily life as well as large-scale commercial exchanges. Automated machines involve many processes in these transactions and have the ability to handle multiple tasks, which are banknote recognition, fitness classification, counterfeit detection, and serial number recognition. Among them, banknote recognition determines the denomination of the input currency paper, and fitness classification evaluates the physical condition of the banknote and decides whether it is suitable for recirculation or if it should be replaced by a new one. The determination of a banknote’s denomination is the primary function of the counting system; meanwhile, fitness classification helps to prevent problems that might occur due to a low quality or damaged banknote being inserted into the system, such as jams or incorrect recognition.

Regarding the functionalities of the automated banknote sorting system, one popular approach is based on image processing, in which input banknotes are captured by various imaging sensors and their optical characteristics are used for classification tasks [1,2]. Previous studies about banknote recognition and banknote fitness classification are mostly reported separately for each of the problems; that is, when dealing with banknote recognition, little research has considered the fitness of the banknotes for recirculation, meanwhile fitness classification studies have mostly been conducted under the assumption that the type and denomination of banknotes have been correctly pre-classified. Considering these issues, this study aims to propose a method to simultaneously solve these two problems.

The structure of our paper is as follows. In Section 2, the related works involving banknote recognition and fitness classification are analyzed and explained in detail. The motivation and contribution of this research is mentioned in Section 3. In Section 4, we explain the proposed banknote type and fitness classification method in detail. The experimental results and conclusions are mentioned in Section 5 and Section 6, respectively. 

## 2. Related Works

The studies concerning banknote recognition of multiple currency types are mostly based on the banknote images captured by visible-light sensors. They can be conducted on either combined or separated countries’ banknote datasets. In the banknote recognition method proposed by Bhurke et al. [3], a color model of the hue, saturation, and value (HSV) of banknote images from five currencies, which were Indian rupees (INR), Australian dollars (AUD), Euros (EUR), Saudi Arabia riyals (SAR), and United States dollars (USD), was used as the features for the template-matching-based recognition system. Banknotes of USD, EUR, and Chinese Renminbi (RMB) were classified in the method proposed by Gai et al. [4] with the combination of quaternion wavelet transform (QWT) and generalized Gaussian density (GGD) for feature extraction and neural network (NN) for classification. Four banknote datasets of United States, South African, Angolan, and Malawian currencies were used for experiments in the banknote recognition methods proposed by Pham et al. [5,6]. In these studies, they used a K-means-based method for recognition of banknotes with the classification features extracted by principal component analysis (PCA) of the discriminative region selected by similarity map [5] and optimized by genetic algorithm (GA) [6] on banknote images. Visible-light banknote images were also popularly used in the studies that simultaneously classify banknotes of multiple currency types. The multinational paper currency recognition methods proposed in Reference [7] were experimented on banknote datasets consisting of four types of currencies from Japan, Italia, Spain, and France. In this research, Takeda et al. [7] employed GA for optimizing the feature extraction and NN for classifying banknotes. Youn et al. [8] proposed a multi-banknotes classification method using multi-templates and correlation matching of five currencies’ banknote images acquired by a contact image sensor (CIS). The convolutional neural network (CNN)-based method proposed by Pham et al. [9] classified banknotes of six national currencies, which were Chinese yuan (CNY), EUR, Japanese yen (JPY), Korean won (KRW), Russian rubles (RUB), and USD, in consideration of the size characteristics of the banknotes. In Reference [10], Hassanpour and Farahabadi employed a hidden Markov model (HMM) for modelling the texture characteristics of banknotes to classify more than 100 denominations of banknotes from different countries.

Regarding fitness classification of banknotes, besides the studies focusing on a certain kind of national currency, there have also been studies considering the variety of currency types [11]. In these studies, experiments were conducted with datasets of banknotes from various countries and regions. Considering soiling as the main reason for unfit banknotes, Lee et al. [12] presented a soiled banknote fitness determination method applied to EUR and RUB based on morphology and Otsu’s [13] thresholding algorithms on banknote images. In the fuzzy-based method proposed by Kwon et al. [14], fitness was determined based on the features extracted from the less textured regions on banknote images captured by visible-light reflection (VR) and near-infrared transmission (NIRT) sensors. This method was tested with the banknote image databases of USD, INR, and KRW. Pham et al. [15] also proposed a CNN-based method for classifying fitness for these three types of currencies using VR banknote images regardless of the denomination and input direction of input banknote into the system. A CNN was also used in the multinational banknote fitness classification method proposed in Reference [11], in which input images to the CNN classifier was the combination of banknote images captured by VR and infrared transmission (IRT) sensors.

Most of the previous works concerning banknote recognition and banknote fitness classification considered only one of these two problems. The banknote recognition studies classified currency papers based on the origin countries, denominations and input directions without considering the physical quality of the banknotes. The ability of rejecting uncertain or misclassification cases in the recognition results was considered in several studies [5,6,16]; however, these studies did not specify whether banknotes were rejected because of low quality or invalid data, and no fitness assessment was proposed in these studies. In the cases of banknote fitness classification studies, most of the works were under the assumption that the denomination and input direction of the banknote being evaluated were known. The CNN-based fitness classification method can determine the fitness levels of a banknote without pre-classification of the banknote image [15] as well as simultaneously for multiple types of currencies [11]. Nevertheless, the fit banknotes may still be rejected if the recognition results of denomination are incorrect. Considering the overall framework of the banknote sorting system, banknote recognition and fitness classification were combined in hierarchical form [2], that is, we needed two classifiers for dealing with these two problems. There have been studies in the field of image classification that have been composed of multiple CNNs in an ensemble and that employed a sum rule score-fusion [17]. Experimental results on various bioimage and medical datasets showed that the ensembles of CNNs outperformed the single CNN trained on a specific dataset [17]. However, an intensive training process was required, and the resulting systems could become very complex. On the evaluation of the state-of-the-art methods, CNNs could be a promising approach for combining banknote recognition and fitness classification based on the advantages of deep-learning techniques, which were comprehensively reviewed in previous researched [18,19]. 

In the CNN-based multinational banknote recognition method proposed in Reference [9], the study focused on the classification of currency type and denomination based on the recognition of the input banknote direction by using visible-light images of banknotes and CNN classifiers. However, the physical quality of the input banknote was not considered, and evaluation of the fitness for recirculation of banknotes was not reported in this study. Considering banknote fitness classification, the method proposed in Reference [14] classifies banknote fitness based on the region of interest (ROI) on banknote images and a fuzzy system. In this study, the whole banknote image was not used for recognition; only certain regions on banknote images were cropped and used for extraction of classification features, which are the average pixel values of ROIs. Since the ROIs on each type of banknote images are different according to the denomination and input direction, this study was conducted on the assumption that the types of banknotes were manually pre-classified. Only two images of each banknote were used for recognition: VR image and NIRT image, which were captured on the same side of the input banknote. The fuzzy-based classifier used in this research does not require a training process; however, it was only applied to classify the fitness of banknotes into two classes of “fit” and “unfit” based on the soiling level on the banknote surface (number of output classes is two). Using deep-learning techniques, the research in Reference [15] aimed at classification of banknote fitness regardless of the denomination and input direction of banknotes belonging to certain currency types. In this study, only one gray-scale image of banknotes captured by VR sensors was used for classification by CNN. Banknotes of INR and KRW were classified into three fitness levels of “fit”, “normal”, and “unfit”, and the number of fitness levels for USD was two. Although the pre-classification of denomination and input directions were not necessary, the experiments were conducted separately on each national currency. In other words, currencies type still had to be manually selected when the number of output classes was two or three. To overcome the issue in Reference [15], the fitness classification method proposed in Reference [11] was able to simultaneously classify fitness of banknotes from three national currencies without pre-classification of currency type, denomination, and input direction of banknotes. A CNN was used as the classifier, and the input images were the combination of three images, which were infrared light transmission IRT and two-side VR images of banknotes captured by three sensors at the same time. In this study, banknotes were classified only into fitness levels without being considered the types of currency, denomination, and input direction (number of output classes was five). 

Based on the analysis of the advantages and disadvantages of the previous related works, we propose a method for classifying both banknote type and fitness using CNN on multi-channel banknote images consisting of VR and IRT images. The problem of a large number of output classes in our proposed method due to multiple currency types and fitness levels can be solved by utilizing the advantage of intensive training process of deep CNN. We also employ additional CNN for estimating fitness values of banknotes using a regression method with quantized average gray values of VR images. The performance of the CNN classifier is evaluated by recognition accuracy, meanwhile the performance of the CNN fitness value estimator is evaluated through the consistency of the estimated values among trials of each banknote by our proposed criterion. Table 1 summarizes the strength and weakness of the previously mentioned studies compared to our proposed method. In Table 2, we give the details of the banknote datasets used for experiments in the previous studies. 

## 3. Contributions

In this study, we automatically classified banknotes in both type (national currency type, denomination, input direction) and fitness level (fit, normal, and unfit), and none of these categories were manually pre-classified (number of output classes is 116). The three channel images of input banknotes composed by IRT and two-side VR images were used with CNN, but we conducted the experiments with various CNN architectures for comparison, rather than using only one CNN architecture as in previous studies, since the number of output classes in this study was huge compared to previous works. Beside fitness classification, we also newly proposed a fitness value estimation of banknote using regression technique of CNN, and using a new criterion of average quantization error (AQE) to evaluate the regression results among trials of input banknotes.

Our proposed method is novel in the following aspects compared to previous works:

- This is the first study on multinational banknote classification of both type and fitness. Considering the related works, each category of banknote type and banknote fitness was reported in the separate studies.
- Our proposed method can simultaneously classify banknote of three types of currency, which are INR, KRW, and USD with three fitness levels in the cases of INR and KRW (namely fit, normal, and unfit) and two levels in the case of USD (namely fit and unfit), into separate classes of currency type, denomination, input direction and fitness levels. To handle with the huge number of output classes, we adopt CNN for the multinational banknote classification of both type and fitness.
- We also estimate the fitness value of input banknote by using CNN regression with the average pixel values of multiple trials of the banknote. For evaluating the estimation results, we considered the consistency of the regression testing results among trials of banknotes, and proposed a criteria called AQE.
- Dongguk Banknote Type and Fitness Database (DF-DB3) and a trained CNN model with algorithms are made available in Reference [20] for fair comparison by other researchers.

## 4. Proposed Method

### 4.1. Overview of the Proposed Method

**Figure 1:**
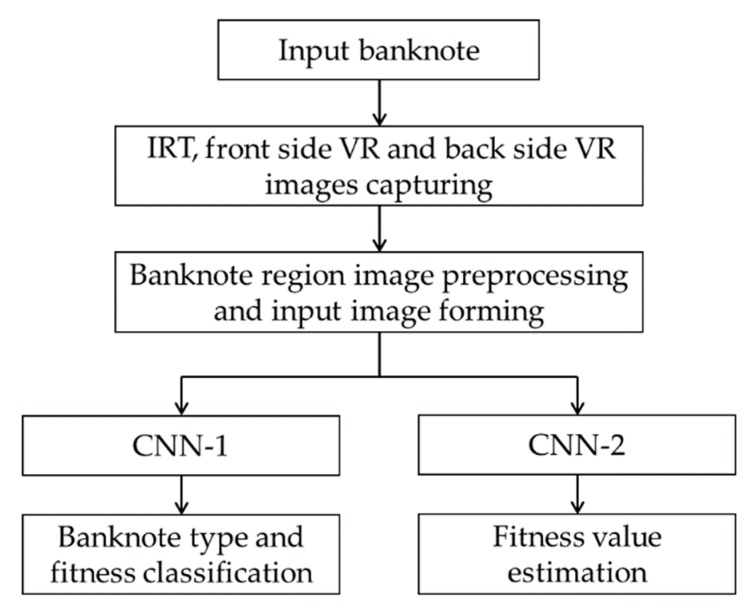
Overall flowchart of the proposed method.

Figure 1 shows the overall flowchart of the proposed method. Banknote images are captured by three sensors which are an IRT and two VR sensors of front and back sides of the banknote. The subsequent preprocessing step including segmentation of the banknote regions from captured images and equally resized to the resolution of 240 × 120 pixels to be inputted to the CNN models in the next steps. Preprocessed banknote images are fed into CNN in the form of a three-channel image, in which the IRT, front side VR, and back side VR images are the first, second, and third channels, respectively. There are two trained CNN models in our proposed system: the first CNN (CNN-1) classifies banknotes for the currency type, denomination, input direction, and fitness level, and the second CNN (CNN-2) estimates the fitness value of the input banknote, as shown in Figure 1.

### 4.2. Acquisition and Preprocessing of Banknote Images

For acquisition of the banknote images in this study, we used capture device including imaging sensors of various wavelengths equipped in the commercial counting machine [21]. IRT and VR images are selected as they can reflect the fitness characteristics of banknotes according to the analysis of the lightning mechanism on new and old banknotes [14]. Each imaging sensor is a line sensor and banknotes images are captured by continuously triggering the line sensor when banknotes are passed through the machine in high speed. The successive captured line images which have the resolution of 1584 pixels are concatenated to form a two-dimensional image of the input banknote. The number of triggering times in the case of INR or KRW for VR and IRT images were 464 and 116, respectively, meanwhile, those in the case of USD were 350 and 175, respectively. Consequently, captured images in the case of USD had the resolutions of 1584 × 350 and 1584 × 175 pixels for VR and IRT images, respectively, and for the case of INR or KRW, the resolutions of VR and IRT images are 1584 × 464 and 1584 × 116 pixels, respectively.

**Figure 2:**
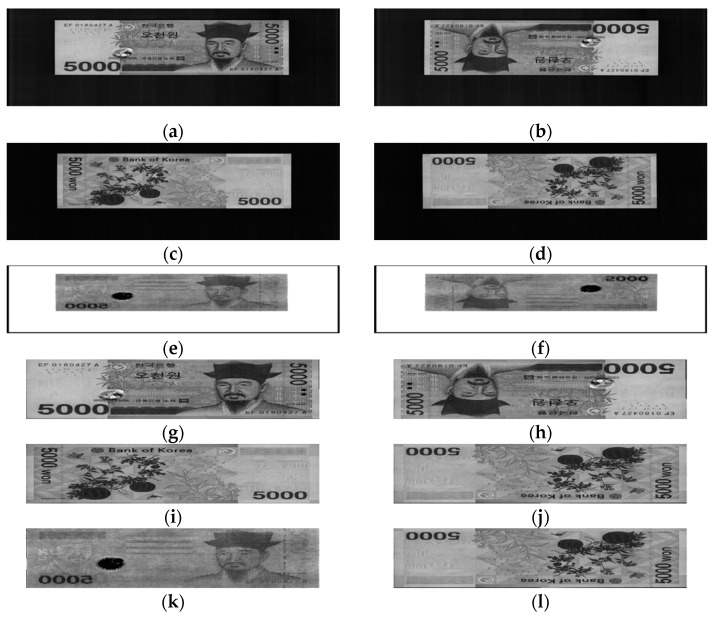
Example of KRW banknote images captured by the device in: (**a**) front side’s forward VR image (A direction); (**b**) front side’s backward VR image (B direction); (**c**) back side’s forward VR image (C direction); (**d**) back side’s backward VR image (D direction); (**e**) forward IRT image and (**f**) backward IRT image; (**g**–**l**) are the corresponding banknote region segmented images from the original images in (**a**–**f**), respectively.

The denomination of banknote is typically determined based on classifying banknote images according to the input direction. Using contact sensors, the input banknote’s image can be captured in one of the four directions, denoted by A, B, C, and D, which are forward front side, backward front size, forward back side, and backward back side, respectively. Examples of VR banknote images in these directions are shown in Figure 2. The capture device simultaneously captures VR images of the input banknote on the front and back side by two sensors denoted by VR1 and VR2, respectively, and IRT image by the sensor from the same side of VR1. Examples of these capture images are shown in Figure 2.

The original captured banknote images consist of the foreground banknote region and background, as shown in Figure 2a–f. We used the built-in segmentation algorithm of the counting machine [15] to extract the banknote region from the captured image in order to remove the redundant information and fix the rotation and displacement of the banknote when being inserted to the machine. The segmented images are then resized to 240 × 120 pixels and combined into a three-channel image composed by IRT, VR1, and VR2 images as the first, second, and third channels, respectively, to be used as the input image to the CNN models in the next steps. Examples of the segmented banknote images are also shown in Figure 2.

### 4.3. CNN Models for Banknote Classification

**Figure 3:**
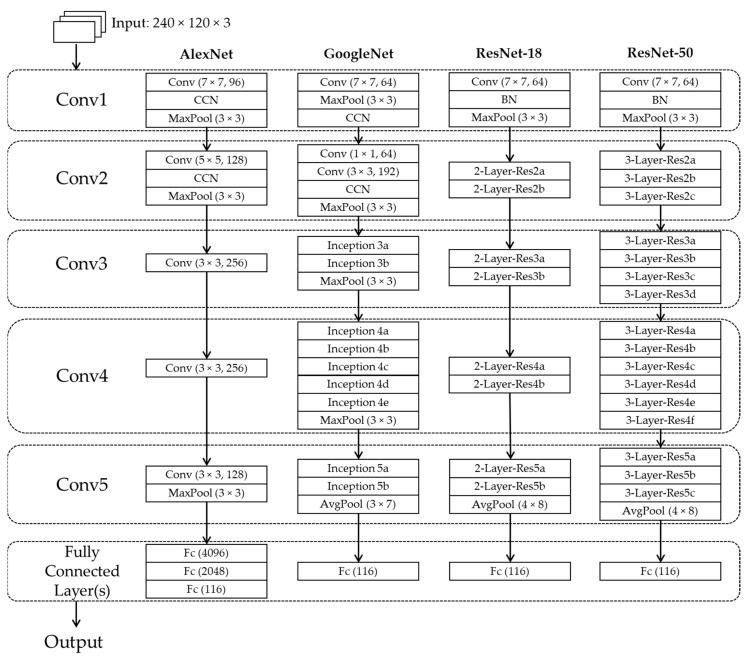
Convolutional neural network (CNN) architectures used for comparisons in our research.

In this research, we classified banknotes from multiple countries according to both banknote types and fitness levels. As a result, the number of output classes is relatively large, compared to the previous works on banknote fitness classification, and the deeper CNN structures should be considered. In our study, we compared the performance of the CNN architectures of AlexNet [22], GoogleNet [23], ResNet-18, and ResNet-50 [24]. Although GoogleNet and ResNets have the deeper structures with more convolutional layers than AlexNet which has only five convolutional layers (denoted by “Conv” in Figure 3), their layers can also be grouped into five convolutional groups, denoted by Conv1 to Conv5, as shown in Figure 3. The original CNN architectures used squared input images of 224 × 224 pixels [22,23,24]; however, the shape of banknotes is typically rectangular. Therefore, we modified the input size of the CNNs structures to 240 × 120 pixels with three channels and trained the modified model from scratch. This helps to not only adapt to the rectangular shape of banknotes but also reduces the numbers of learnable parameters in the networks. The detail architectures of the CNNs with each layer’s attributes and output feature map size are given in Table 3, Table 4, Table 5 and Table 6.

In all of the CNN structures, rectified linear units (ReLUs) are passed through each convolutional layer and the fully-connected layers. This activation function is popularly used in CNN as it works as a rectifier that allows only the positive values to pass and helps to improve training speed as well as avoid the gradient-vanishing, compared to the conventional sigmoid function [11,25]. Local response normalization units are used in all the CNN architectures to aid generalization. In the cases of AlexNet and GoogleNet, cross-channel normalization (CCN) is used at the first two convolutional layers [22,23]. In the cases of ResNets, batch normalization (BN) that allows speeding up training of CNN and reduce the sensitivity to network initialization is used in the first convolutional layers and before ReLU units [24,26]. The formula of CCN and BN are shown in the following Equations (1) and (2), respectively.

(1) $$\bar{x}=\frac{x}{{\left(K+\frac{\alpha \cdot S_{Sqr}}{WindowChannelSize}\right)}^{\beta }}$$

(2) $$\bar{x}=\gamma \left(\frac{x-\mu_{B}}{\sigma_{B}}\right)+\delta$$

where *x* is the mini-batch data or the activity of the kernel, $\bar{x}$ is the value obtained by normalization. In CCN, *K*, *α*, and *β* are the hyperparameters, *S_Sqr_* is the sum of the squared elements in the normalization window with the size defined by *WindowChannelSize* argument. The parameters of *WindowChannelSize*, *K*, *α,* and *β* for CCN are chosen to be 5, 1, 10^−4^, and 0.75, respectively [11]. In BN, *µ_B_* and *σ_B_* are the mean and square-root of the variance over a mini-batch and over each input channel, respectively; *γ* and *δ* are the learnable scale factor and offset values, respectively.

**Figure 4:**
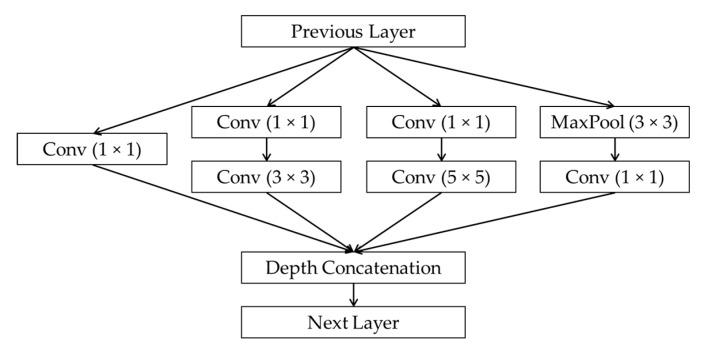
Structure of the Inception module.

The GoogleNet architecture consists of the convolutional blocks called Inception modules [23], denoted by “Inception” in Figure 3. The structure of Inception module is shown in Figure 4. The idea of Inception structure is to perform convolution on an input with three different sizes of filters (1 × 1, 3 × 3, and 5 × 5) to extract the features from the meaningful details of various scales on images. With the 1 × 1 convolutional layers in each branch, the computational complexity can be reduced before the 3 × 3 and 5 × 5 convolutions [23]. The output feature maps of the filter branches are concatenated along the third dimension at the output of the Inception block. The numbers of filters in the Inception modules of the GoogleNet architecture are varied according to the convolutional groups, and the size of the feature maps at the output of each group is shown in Table 4.

**Figure 5:**
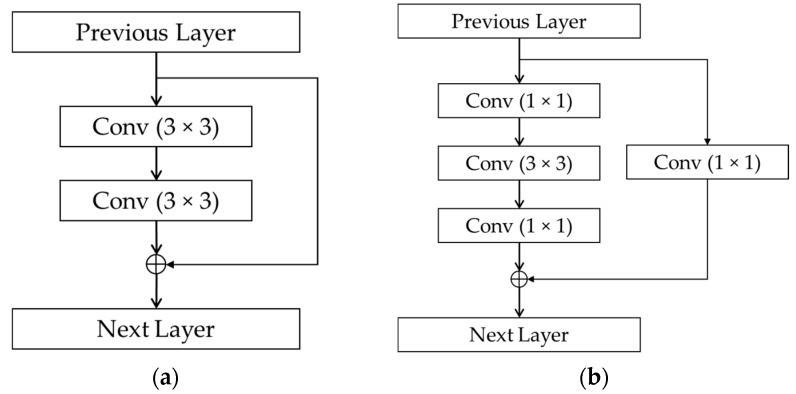
Structures of residual blocks: (**a**) two-layer deep block; (**b**) three-layer deep (bottleneck) block.

In the ResNets architectures, the residual blocks are introduced with either two-layer depth used in ResNet-18 (denoted by “2-Layer-Res” in Figure 3) or three-layer depth used in ResNet-50 (denoted by “3-Layer-Res” in Figure 3), as shown in Figure 5. These structures were proposed in Reference [24] to solve the problem of degradation in the performance when the network depth increases, that is, when deep networks starts converging, with the increment of the network depth, accuracy becomes saturated and then degrades. The worst case happens when the deeper network layers act as an identity function where the input and output are equal. If the identity function is optimal, the shortcut connections can be used either directly when the input and output are of the same dimension (Figure 5a), or with the projection by using 1 × 1 convolution for dimensional matching (Figure 5b) [24], and the output of the residual block is the element-wise summation of the feature map of two or three-layer convolutional block and that delivered from the input by the skip connection. The details architectures of ResNet-18 and ResNet-50 with 240 × 120 × 3-pixel input images used in our study are described in Table 5 and Table 6, respectively.

The convolutional layers are finally connected to the fully connected layers, which act as the classifier in the CNN architectures, by passing the feature map through a pooling layer. In the case of AlexNet, max pooling was used, meanwhile in the case of the remaining CNN structures, average pooling was used, as shown in Table 3, Table 4, Table 5 and Table 6. There are three fully-connected layers denoted by “Fc1” to “Fc3” in Table 3 of the AlexNet architecture, and only one fully-connected layer in each case of GoogleNet, ResNet-18, and ResNet-50, denoted by “Fc” in Table 4, Table 5 and Table 6, respectively. In the connections before the fully-connected layers of AlexNet and GoogleNet, a dropout layer was employed to prevent overfitting in the training process of the network. This is a regularization method that randomly disconnects the network nodes during training [27]. In this study, the probability of a network connection to be dropped out by this method was chosen to be 50% [11,22].

In the classification CNN, denoted by CNN-1 in Figure 1, the number of output nodes is the same with the number of classes. In this study, banknotes are classified in both types of currency, denomination and input direction as well as fitness levels. In the INR dataset there are six denominations, captured in two input directions with three levels of fit, normal, and unfit. Totally there were six denominations × 2 directions × 3 fitness levels = 36 classes of INR banknotes. In the KRW dataset, there were two denominations with the similar three fitness levels, and two denominations with two levels of fit and normal, all were captured in four directions, resulting to 40 classes of banknotes in this dataset. The remaining USD dataset consists of five denominations of banknotes inputted in four directions, and two fitness levels of each kinds. There are consequently 40 classes of banknote type and fitness in USD dataset. Totally there were 116 (36 + 40 + 40) classes in the banknote dataset used in our study. From the output *y_i_* of the *i*th node in the last fully connected layer consisting of *N* nodes, the input banknote was considered to have the probability *p_i_* of belonging to the *i*th class by using the normalized exponential function (softmax function) [9,22]. The Softmax function is described as the following in Equation (3).

(3) $$p_{i}=\frac{e^{y_{i}}}{\sum_{i=1}^{N}e^{y_{i}}}$$

### 4.4. Banknote Fitness Value Estimation by CNN Regression

In the case of CNN-2 used for fitness value estimation, as shown in Figure 1, the classification output layer in the architecture of the corresponding CNN-1 was replaced by the regression layer which has only one output. The loss function was also replaced in the regression training process, from the cross-entropy loss used in CNN-1 training into the half-mean-squared-error loss for the CNN-2 training. With *N* samples of training data, *K* output classes, the loss functions of cross-entropy and half-mean-squared-error used in CNN classification and regression were calculated as the following Equations (4) and (5), respectively [28]:

(4) $$L_{CE}=-\sum_{i=1}^{N}\sum_{j=1}^{K}t_{ij}\ln y_{ij}$$

(5) $$L_{HMSE}=\frac{1}{2}\sum_{i=1}^{N}\frac{{\left(t_{i}-y_{i}\right)}^{2}}{N}$$

where *L_CE_* and *L_HMSE_* are cross-entropy loss and half-mean-squared-error loss, respectively. In (1), *t_ij_* is the indicator representing the belonging of *i*th sample to the *j*th class, *y_ij_* is the output of *i*th sample for *j*th class. In (2), *t_i_* and *y_i_* are the target output and the network’s prediction value of *i*th sample, respectively. 

**Figure 6:**
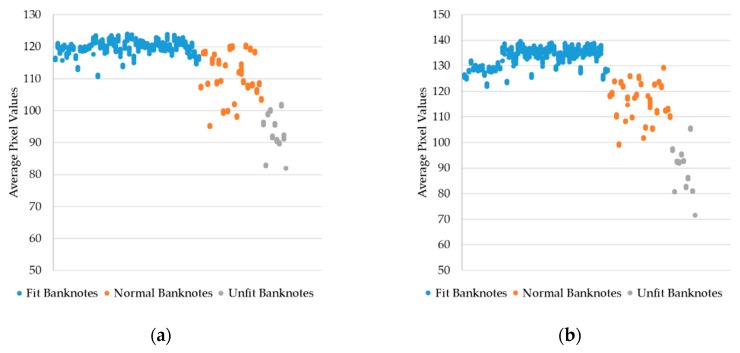

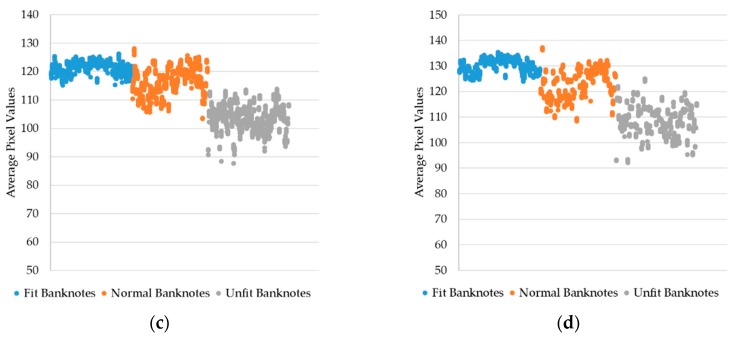
Comparison of average pixel values of VR banknote images from (**a**) INR10 front forward direction (INR10A); (**b**) back side of banknotes in (**a**) (INR10C); (**c**) KRW5000 front backward direction (KRW5000B); and (**d**) back side of banknotes in (**c**) (KRW5000D).

In the dataset used in our research, each banknote of KRW and INR was inserted into the capturing device for multiple times. As a result, the banknote image datasets of KRW and INR consist of images that represent multiple trials of the banknotes. In this study, we proposed the fitness value estimation method that procedures the unique result for each banknote regardless of the trials. The reference target values for fitness values regression were calculated by averaging the pixel values of the VR trial images that belonged to each of the input banknotes. The VR images were chosen because they show good reflection of the fitness characteristic of banknotes as analyzed in previous studies [14,15]. Here, we expect that the average pixel values of unfit banknote images are smaller than those of the fit banknote images, as the VR images tend to become darker when the banknotes’ quality degrades [14]. This can be confirmed by the scatter plots of the examples of average VR1 and VR2 pixel values of banknotes from the same types (denomination and direction) as in Figure 6. It can be seen from the Figure 6 that there are relative differences in the average brightness of the VR banknote images of both sides among three levels of fitness. As a result, average pixel values of VR images were used for extracting the reference target values of CNN regression.

When we obtained all of the average VR trial image pixel values of the banknotes in the training dataset, we perform min–max scaling and rounding (quantization) on the obtained values to have the integer values of 1 to 10 and reassign these quantized values to the sample images as the target output fitness values. This quantization task is performed separately according to banknote types of denomination and input direction, because the patterns and brightness of VR images are different among types of banknotes. Using the integer values for fitness value also helps on evaluation of the estimation results’ consistency, in which, if the fitness values are integer number in the range of 1 to 10, in other words, quantized to have the discrete fitness level from 1 (the most unfit) to 10 (the most fit), the estimated values from the trials of a banknote should distribute in as few quantization levels as possible. The criterion for evaluating the performance of the fitness value estimation will be explained in the next section with experimental results. 

## 5. Experimental Results

### 5.1. The Experimental Banknote Image Dataset

**Figure 7:**
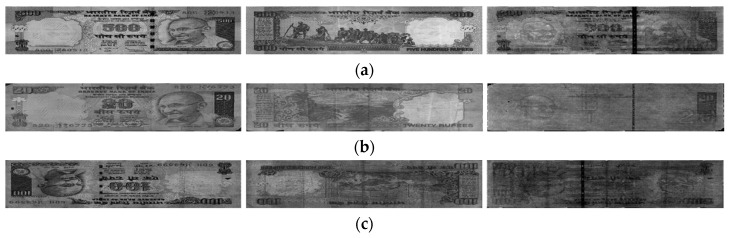
Examples of INR banknote images: (**a**) fit; (**b**) normal; and (**c**) unfit banknotes. From left to right of each figure are the VR1, VR2, and IRT images of the input banknote, respectively.

**Figure 8:**
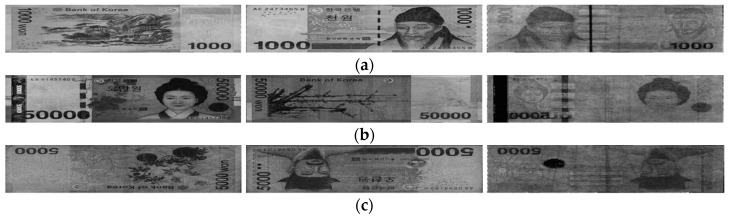
Examples of KRW banknote images: (**a**) fit; (**b**) normal; and (**c**) unfit banknotes. Images in each figure have the same order as those in Figure 7.

**Figure 9:**
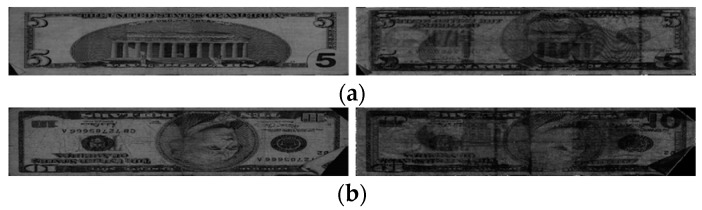
Examples of USD banknote images: (**a**) fit and (**b**) unfit banknotes. Images on the left and right of each figure are the VR and IRT images of the input banknote, respectively.

In this study, we used the multinational banknote image dataset composed of banknotes from three national currencies, which are INR, KRW, and USD. In the INR dataset there were six denominations including 10-, 20-, 50-, 100-, 500-, and 1000-rupee banknotes with three levels of fitness of fit, normal, and unfit. In KRW, banknotes with the same three levels of fitness were collected from two denominations of 1000 and 5000 won, and those with two levels of fit and normal were collected for KRW 10,000 and KRW 50,000. In the remaining dataset of USD, two levels of fit and unfit were defined for five denomination of 5, 10, 20, 50, and 100 dollars. Examples of banknote images in each dataset with different fitness levels are shown in Figure 7, Figure 8 and Figure 9. In the cases of INR and KRW, input banknote images consisted of three images: two VR images captured on both front and back sides of the banknote, denoted by VR1 and VR2 images, respectively, and one IRT image captured from the same side of VR1. The number of images per banknote in the case of USD was two images, which were VR and IRT captured from the same side. By this capturing method, the number of input banknotes in the dataset was equal to the number or IRT images in all three types of currency. We combined these captured banknote images into a three-channel image, in which the first, second, and third channels are the IRT, VR1, and VR2 of the input banknote, to be inputted to the CNN for classification. In the case of USD, the VR images were duplicated in the second and third channel of the input image to adapt to the CNN model. The duplication of VR images for the second and third channels of USD input images may not be the best solution. In order to compensate the lack of VR2 images in the case of USD, we can consider the approach of image synthesis techniques such as using PCA [29] or using the generative adversarial networks (GANs) [30,31]. However, image generation is not the main purpose of this research and can be considered for the future works. The number of input banknotes in each fitness levels of each type of currency and denomination are shown in Table 7. This dataset is available as DF-DB3 in Reference [20]. Including the denominations, input directions and fitness levels of banknotes, there are totally 116 classes in our experimental multinational banknote type and fitness database.

### 5.2. Training and Testing for Banknote Type and Fitness Classification

In the first experiments of training and testing for banknote type and fitness classification using CNN or CNN-1 in Figure 1, we performed the two-fold cross-validation. For this purpose, we randomly divided the banknote dataset into two parts, used one for training and another one for testing, and we repeated the process with these two parts swapped. The overall performance was evaluated by calculating the average classification accuracy of the two testing results. 

There are four CNN architectures used for experiments in this study: AlexNet, GoogleNet, ResNet-18, and ResNet-50 [22,23,24], as mentioned in Section 4.3. ResNet-50 was tested in the comparative experiments in the previous study [11]. We further tested with the shallower version of ResNet, which is ResNet-18. The other CNN architectures such as VGG-16, VGG-19 [32], and ResNet-101 were taken into account. However, due to the large number of learnable parameters in these network architectures [33], the models were not able to be trained on our system and an “Out of Memory” error was thrown back. As a result, we tested the performance of our method with the four mentioned CNN architectures. We modified the image input layers of these architecture to the size of 240 × 120 × 3 to adapt the rectangular shapes of banknotes and train the CNN models from scratch. As a result, intensive training was required, and we performed data augmentation to generalize the training data and reduce overfitting [9]. The data augmentation method was based on boundary cropping of the training images [9,11,15], that is, we randomly cropped images of the training dataset in the range of 1 to 10 pixels on the four boundaries. The multiplication factors were varied according to the classes of banknotes so that the numbers of training data were increased to be relatively comparable among classes, as shown in Table 7. Our training and testing experiments were conducted by using the MATLAB implementation of CNN [34] on a desktop computer with the following configuration: Intel® Core™ i7-3770K CPU @ 3.50 GHz [35], 16 GB DDR3 memory, and NVIDIA GeForce GTX 1070 graphics card (1920 CUDA cores, 8 GB GDDR5 memory) [36]. The training from scratch method was the stochastic gradient descend (SGD), which updates the network parameters on batches of data points at a time [28], with 150 training epochs for GoogleNet and 100 epochs for the remaining architectures, learning rate of each architectures of AlexNet, GoogleNet, ResNet-18, and ResNet50 initiated at 0.01, 0.001, 0.01, and 0.1, respectively, and reduced by 0.1 times at every 20 epochs. We also conducted the experiments of transfer learning [34] with the pretrained CNN models by ImageNet database of the same architectures [33] and compared the testing results with those obtained by CNN models trained from scratch in this study. In the training process of transfer learning, the number of training epochs was 15 and learning rate was 0.001. 

**Figure 10:**
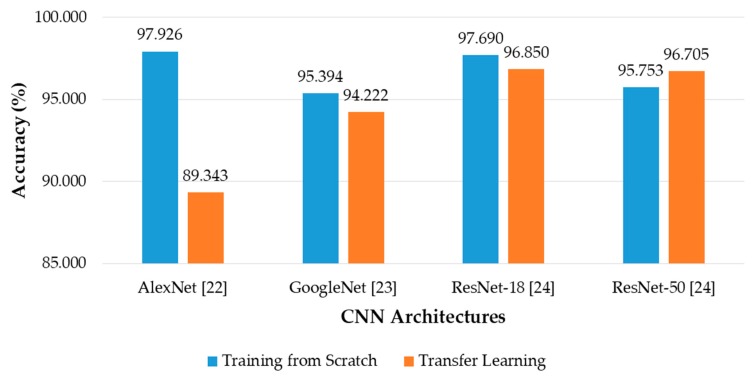
Average accuracy of banknote type and fitness classification using various CNN architectures with training from scratch and transfer learning.

In the next testing experiments, we performed the classification with the trained CNN models on the remaining subsets in the two-fold cross-validation. From the obtained accuracies of the two testing subsets, we calculated the average accuracy of the CNN classifier by taking the ratio of correctly recognized cases and total number of samples as the following formula [37]:

(6) $$Acc=\frac{N_{GA2}+N_{GA1}}{N}$$

where *N_GA_*_1_ and *N_GA_*_2_ are the number of accurately classified (genuine acceptance) cases of the first and second testing results in the two-fold cross-validation, *N* is the total number of banknotes in the dataset, and *Acc* is the average classification accuracy. Figure 10 shows the results of average classification accuracy for each case of CNN architecture, with both the models training from scratch or transfer learning. 

**Figure 11:**
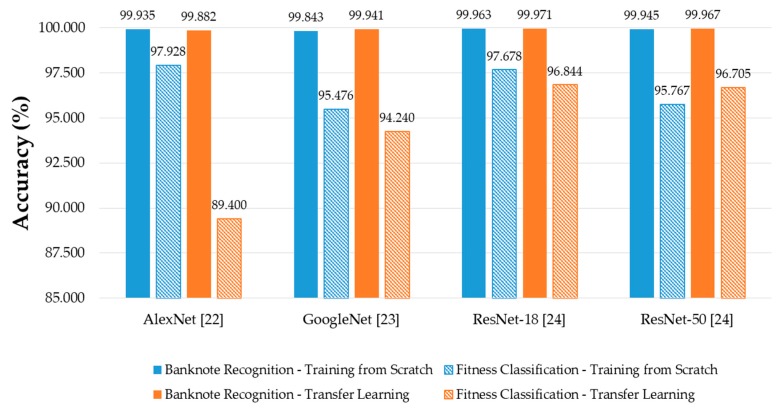
Average accuracy calculated separately for banknote recognition and fitness classification using various CNN architectures with training from scratch and transfer learning.

It can be seen from the Figure 10 that in most of the cases, CNN models trained from scratch yielded better results than those trained by transfer learning. This can be explained first by the difference in the input image size of the CNN models trained from scratch and that of the pretrained CNN models. By using the size of 240 × 120 × 3 pixels, the input images reflect better the rectangular shape of banknotes than those with square shape in the cases of pretrained CNNs. In this study, the rectangular banknote images are reshaped to squared images with the size of 224 × 224 × 3 pixels to be fed to the pretrained CNN models by bilinear interpolation algorithm. The unevenness of the average testing accuracies between training from scratch and transfer learning was smaller in the cases of deep networks than that in the case of the shallow network of AlexNet, the reason for this result is as follows: the classifier parts of the deep networks consist of only one fully-connected layer, meanwhile there are three fully-connected layers in the case of AlexNet, as shown in Figure 3. As a result, through intensive training from scratch with banknote image dataset, the classifier part of AlexNet-based proposed method performed better than that of the pretrained AlexNet model that was trained by the ImageNet database [33]. The very deep network architectures such as GoogleNet and ResNet-50 gave less accuracies than AlexNet and ResNet-18, because the deep networks typically required more intense training task. We further investigated the average accuracy with the certain tasks of the system, which are banknote type recognition and banknote fitness classification, with the obtained results. For calculating these results, besides the genuine acceptance cases for both banknote type and fitness classification, we considered the cases that although banknote fitness was misclassified but banknote type was correctly recognized as the genuine acceptance cases of banknote types, and vice versa as the genuine acceptance cases of banknote fitness. With the obtained number of genuine acceptance cases for both categories, average accuracies were calculated as in Equation (6). The results are shown in Figure 11.

**Figure 12:**
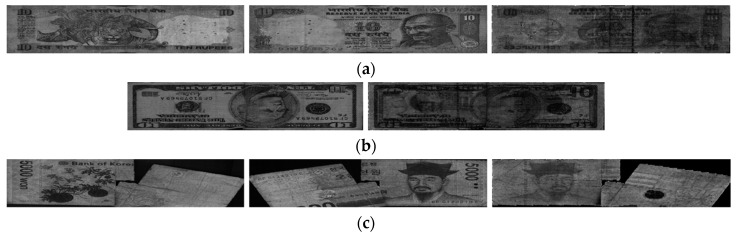
Examples of error cases in the testing of our proposed method: (**a**) Case 1—unfit banknote with banknote type correctly recognized but fitness level misclassified to normal; (**b**) Case 2—unfit banknote with misclassified banknote type; and (**c**) Case 3—misclassification of both banknote type and fitness. In (**a**) and (**c**), images were arranged similarly as those in Figure 7; in (**b**), images were arranged similarly as those in Figure 9.

It can be seen from Figure 11 that the error rates of the overall results in Figure 10 were mostly caused by the error in the fitness classification task of the system. Although the banknote recognition results were slightly better in the cases of ResNets, the fitness classification results were still the best in the case of AlexNet. Moreover, the difference between the accuracies of banknote recognition and fitness classification in the case of AlexNet was the smallest among the compared CNN architectures, where the second smallest was that of ResNet-18, and both were the models fully trained by our banknote image database. The results can be explained by the fact that the network architectures with a smaller number of layers tend to perform better with a smaller dataset when training processes are performed from scratch [38], especially when the training dataset has significant differences from that used for pretraining the transfer learning models; in this case, they were our banknote fitness dataset and the ImageNet database [33]. Since the difference between these two shallow networks were not significant in both tasks of banknote recognition and fitness classification, and we chose either AlexNet or ResNet-18 to modify to have rectangular input images of 240 × 120 × 3 as the CNN model for our proposed method. In Figure 12, we show the examples of error cases occurring in the testing process by our proposed method.

In the first case of Figure 12a, the input banknote was unfit due to the stains which can be visible on banknote images, but the overall brightness of the VR1 was slightly high. Consequently, the banknote was misclassified to normal. In the second case of the USD image in Figure 12b, the banknote was correctly classified as unfit, but the denomination was incorrectly recognized, as the quality of the banknote affected the recognition result. In the last case of Figure 12c, the banknote was so severely damaged that both the banknote type and fitness could not be recognized.

To test the difference in the classification results when the segmentation of the banknote region from the original captured image was not used, we conducted comparative experiments with two-fold cross-validation on the original captured banknote images, whose examples are shown in Figure 2a–f. The original banknote images which include both banknote regions and background were equally resized to 240 × 120 pixels and combined into a three-channel image in the order of IRT, VR1, and VR2 images from first to third channels to be subsequently inputted to the CNN models. In these comparative experiments, we tested the performance of using unsegmented banknote images with two CNN architectures, which were AlexNet and ResNet-18 trained from scratch, as they gave better results compared to the remaining architectures, as shown in Figure 10. The comparative experimental results of the average classification accuracies with two-fold cross-validation are shown in Table 8.

The results given in Table 8 show that the segmentation step helped to boost the performance of the proposed method, in terms of higher average classification accuracies compared to those when not using banknote region segmentation. The reason for the experimental results can be explained as follows. From the captured banknote images, the segmentation process extracts the banknote regions which contain information of textures and patterns for classifying and removing redundant data from the background. Moreover, the original captured images were also affected by rotation and displacement of the banknotes when being inserted into the machine. As a result, the banknote regions captured by various sensors cannot be aligned when being arranged into the three-channel input image of CNN. When the banknote region segmentation was adopted, only the meaningful information from banknote images were used for training the CNN models, and the trained filters responded properly to the aligned banknote regions, which consequently helps to enhance the testing accuracy and overall performance of the proposed method, as shown in Table 8.

**Figure 13:**
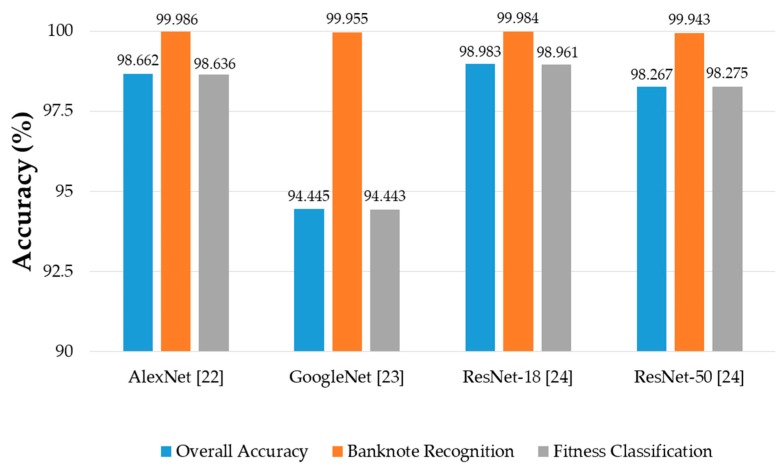
Average testing accuracy of five-fold cross-validation with overall accuracy and accuracy calculated separately for banknote recognition and fitness classification using various CNN architectures with models trained from scratch.

For further confirm the generalization of the classification results of the CNN models trained from scratch, we conducted the additional experiments with five-fold cross-validation. For this purpose, we randomly divided the dataset into five subsets, used the combined four subsets for training and the remainder for testing. This process of training and testing was repeated five times with the subsets alternated. We subsequently calculated the average classification accuracies of the four CNN architectures with the models trained from scratch. The experimental results are given in Figure 13. 

The results given in Figure 13 shows that the average classification results were much enhanced in the cases of AlexNet and ResNet-18 and outperformed the remaining CNN structures with five-fold cross-validation, as the training tasks in the five-fold cross-validation were more intensive. The more intensive training tasks also helped to increase the fitness classification results in the case of ResNet-18, consequently enhancing the overall classification accuracy compared to the other CNN architectures, as shown in Figure 13.

Once the CNN structure for banknote type and fitness classification (CNN-1) was determined, we proceed to the experiments for the estimation of banknote fitness value using CNN regression (CNN-2) in the next section.

### 5.3. Training and Testing for Banknote Fitness Value Estimation with CNN Regression

In the training and testing experiments for the second CNN which estimated the fitness value of banknotes (CNN-2 in Figure 1), we removed the last classification layer of the CNN structure used for banknote type and fitness classification (CNN-1) and replaced it with the regression layer that consisted of only one output. The regression CNN models were trained with the reference target output values calculated by quantization of the average VR image pixel values, as mentioned in Section 4.4. These target output values had a range of integer numbers from 1 to 10, corresponding to the increment of fitness of banknotes in the separate types of denomination and input direction.

The banknote datasets of INR and KRW composed of banknote images captured from input banknotes that were inserted multiple times to the system, that is, there were multiple trials of banknotes in the cases of INR and KRW. We evaluated the performance of the estimation task by not only calculating the difference between desired and predicted output values but also assessing the consistency of the estimated values among trials of a banknote. For this purpose, we performed two-fold cross-validation on the banknote dataset with CNN regression using the same four CNN architectures that were previously used for banknote classification with the same data augmentation method. In the experiments of CNN regression training, number of training epochs for AlexNet was 100, and that for the other architectures were 60, learning rate for all the architectures was initially set to 0.001, and reduced 10% every 20 epochs. In the CNN fine-tuning experiments, we set the number of epochs and learning rate to 15 and 0.001, respectively, for all the pretrained models. 

**Figure 14:**
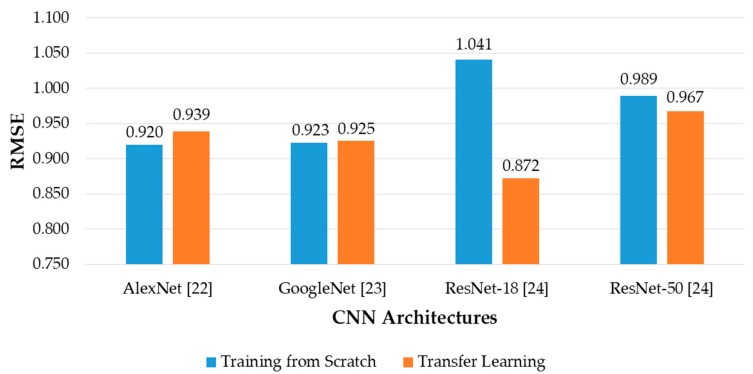
Average root-mean-squared error (RMSE) of banknote fitness estimation using various regression CNN architectures with training from scratch and transfer learning.

In the experiments of fitness level estimation, we used the remaining subsets of the banknote database that were not used for training in the two-fold cross-validation method to test the performance of CNN regression. The first criterion to be calculated was root-mean-squared error (RMSE) between predicted integer fitness value and the desired value. The calculation results of RMSE are shown in Figure 14 for each CNN architecture with both training from scratch and transfer leaning.

**Figure 15:**
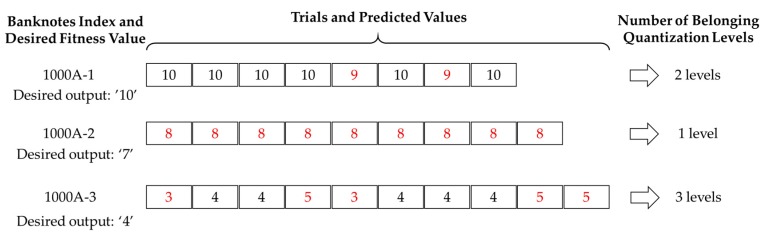
Examples of input banknotes with desired fitness values and their trials with predicted fitness values.

Although the best regression testing result was obtained from ResNet-18 with transfer learning, in term of low RMSE, it was difficult to determine whether the predicted values obtained from trials of a certain banknotes in the dataset were similar to each other, in other words, the consistency of the predicted results should be determined. For this purpose, we proposed a criterion to evaluate the difference in the estimated fitness values among trials of a banknotes, called quantization error (QE), as the predicted values were integers, then we calculate the average quantization error of the results obtained from entire dataset. The basis of calculation process is illustrated in Figure 15. 

**Figure 16:**
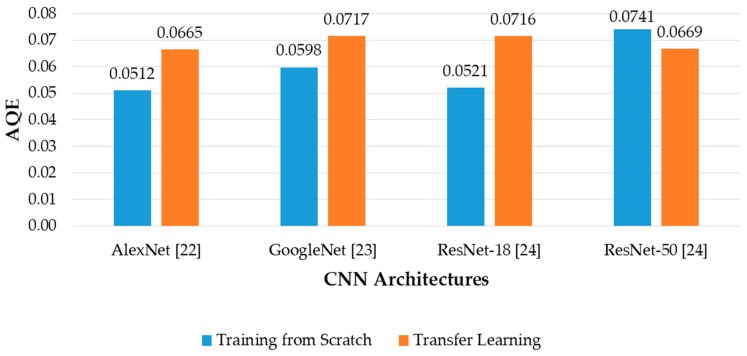
Average quantization error (AQE) of banknote fitness estimation using various regression CNN architectures with training from scratch and transfer learning.

In Figure 15, it was assumed that we had three banknotes from a national currency with denominations of 1000 inserted into the system several times, where each of these banknotes were input from the front side, forward direction (denoted by 1000A-1 to 1000A-3). The examples of the desired fitness value of each banknote and predicted values by CNN regression of each trial of the banknote are shown in Figure 15. In the first case of 1000A-1, for most of the cases, fitness values were correctly estimated and there were only two error cases, where differences from the desired values was 1. In the second case of 1000A-2, all the trials were predicted to have higher fitness values than expected; however, the results were the same among trials. In this case, the consistency of the estimated results was assured. In the last case, the predicted results spread to three levels, this shows the worst consistency among three examples. The QE of each banknote was calculated as follow:

(7) $$\mathrm{QE}=\{\begin{array}{l} \frac{N_{Q}-1}{N_{T}-1}\;\mathrm{if}\;N_{T}>1\;\\ \;0\;\mathrm{if}\;N_{T}=1 \end{array}$$

where *N_Q_* is the number of belonging quantization levels of the trials’ predicted values, *N_T_* is the number of trials per banknote. The QE had values in the range of 0 to 1, in which zero corresponds to the best case (no quantization error, or all the predicted results have the same value) and 1 corresponds to the worst case when all the trials have predicted values different from each other (*N_Q_* = *N_T_*). From Figure 15, the QE of the 1000A-1 banknote was equal to (2 − 1)/(8 − 1) = 0.142, in the case of 1000A-2 it was zero, which was the best case, and the worst among the three examples was that of 1000A-3 with the QE = 0.222. With the QE obtained from each banknote’s trials, the average quantization error (AQE) of the estimated results of the entire dataset was calculated by taking the weighted average of QEs with number of trials per banknote as follow:

(8) $$\mathrm{AQE}=\frac{\sum_{i=1}^{M}N_{Ti}{\mathrm{QE}}_{i}}{\sum_{i=1}^{M}N_{Ti}}$$

where QE*_i_* and *N_Ti_* are quantization error and number of trials of the *i*th banknote in the dataset, *M* is the total number of datasets. The denominator of Equation (8) is the total number of images in the dataset. Figure 16 shows the comparison of AQE among fitness level estimation results of the CNN architectures, with both training from scratch and transfer learning.

**Figure 17:**
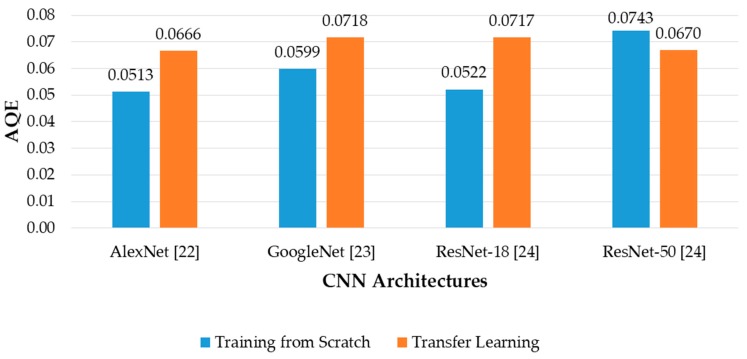
Adjusted average quantization error (AQE) of banknote fitness estimations using various regression CNN architectures with training from scratch and transfer learning with the differences of estimated trial values among banknotes considered.

The QE determination described above does not take into account the cases where the predicted values were significantly different among trials of a banknote. That is, *N_Q_* in Equation (7) treats these cases the same with the cases where the differences are only one level. To overcome this drawback, we considered the range, i.e., the difference between the maximum and minimum of the estimated values among the trials of a banknote, and took the geometric average with the numerator of the QE formula in Equation (7). The adjusted QE of a banknote, denoted by QE*_A_* is computed as follow:

(9) $${\mathrm{QE}}_{A}=\{\begin{array}{c} \frac{\sqrt{\left(N_{Q}-1\right)R}}{N_{T}-1}\;\mathrm{if}\;N_{T}>1\;\\ 0\;\mathrm{if}\;N_{T}=1 \end{array}$$

where *R* is the range of the estimated values of all the trial of the banknote. The remaining symbols denote similar to those in Equation (7). If the differences among trials of a banknote are only one step from each other, the value of *R* is equal to (*N_Q_* − 1), and the numerator of the Equation (9) becomes equal to that of Equation (7). This case can be seen in the examples of Figure 15 as either the cases of 1000A-1 or 1000A-3 banknotes, where the R values were 1 and 2, equal to 2 levels minus 1 and 3 levels minus 1 in the corresponding cases, respectively. If the differences among trials are larger, the value of *R* increases and the QE of the banknote in such a case is larger than that of the case with smaller differences among trials. With the adjusted QE, we calculated the AQE of the estimated results of the dataset by replacing QE with QE*_A_* in the weighted average of Equation (8) and the results are shown in Figure 17.

The results in Figure 16 and Figure 17 show that the proposed method using AlexNet trained from scratch with modified input image size produced the best results based on the analysis of consistency of estimated fitness values among trials of input banknotes with AQE criterion. The differences between originally proposed and adjusted AQE results are not notable in all the cases, because the estimated value of trials of each banknote in the dataset is not significantly different from each other. When the range value is considered in the QE calculation, it did not much affect the overall AQE result. In terms of low AQE, the best and second-best fitness estimation results were obtained from AlexNet and ResNet-18 trained from scratch, similar to those in the banknote classification experiments in the previous section, as shown in Figure 10. As a result, we can conclude that the proposed CNN architecture based on ResNet-18 and AlexNet can be respectively used for classification of banknotes and estimation of fitness value, as the obtained results outperformed the other architectures, in terms of higher average classification accuracy and lower estimation error among trials of banknotes.

In order to further compare the performance of using our proposed method to that using the previously reported methods in References [9,15] for recognition of both type and fitness of banknotes, we conducted additional experiments on our database. Referring to References [9,15], we employed AlexNet CNN architecture with input VR images for classification of banknotes in both type and fitness level, which were reported separately in these previous works [9,15]. This method of selecting input banknote images and CNN architecture was also used for estimating banknote fitness values in the comparative experiments to our proposed estimation method. The comparative experimental results of our proposed method and the previous method with average accuracies and estimation errors are shown in Table 9.

It can be seen from Table 9 that the proposed method outperformed the previous method in term of higher banknote classification accuracy and lower fitness value estimation error. The reason for the comparative experimental results can be explained by the inclusion of the IRT banknote images for classification and estimation of banknote fitness. The use of various sensors of visible-light and near-infrared for capturing banknote images enabled the system to collect useful optical information of banknotes in various wavelengths. This helped to increase the fitness classification accuracy and consequently enhance the overall classification accuracy and reduce the fitness value estimation error, as shown in Table 9.

## 6. Conclusions

In this study, we proposed a banknote classification method that simultaneously classifies banknotes from multiple national currencies in both types and fitness levels using the combination of IRT and VR images of the input banknote and the CNN. We also designed a CNN-based estimator of banknote fitness value based on the regression of CNN with scaled average VR image pixel values of banknotes. To evaluate the performance of the estimator, we proposed the criterion of AQE which is used for determining the consistency of the fitness estimated values among trials of input banknotes. The experimental results using two-fold cross-validation on the combined banknote image dataset of INR, KRW, and USD banknotes showed that our proposed method yields good classification and estimation performance and outperformed the other methods with various CNN architectures, in term of higher classification accuracy and more consistent estimated values among banknotes’ trials. For future works, we planned to combine the other functionalities in the banknote sorting domain, such as counterfeit detection or serial number recognition, to our system, as well as conduct our research with various types of physical currencies. We also consider employing an ensemble of CNN models for combining these banknote classification tasks and boost the overall performance of the system.

## Figures and Tables

**Table 1:** Summary of related works on banknote recognition and fitness classification considering the variety of currency types.

Category		Method	Advantage	Disadvantage
Banknote recognition	Single Currency Recognition	- Using HSV color features and template matching [3]. - Using QWT, GGD feature extraction, and NN classifier [4]. - Using similarity map, PCA, and K-means-based classifier [5,6].	Simple in image acquisition and classification as recognition process is conducted on separated currency types with visible light image.	Currency types need to be manually selected before recognition.
	Multiple Currency Recognition	- Using GA for optimizing feature extraction and NN for classifying [7]. - Using multi-templates and correlation matching [8]. - Using CNN [9]. - Using HMM for modeling banknote texture characteristic [10].	Multinational banknote classification methods do not require the pre-selection of currency type.	Classification task becomes complex as the number of classes increase.
Banknote fitness classification	Using Single Sensor	- Using image morphology and Otsu’s thresholding [12]. - Using CNN with VR images [15].	Simple in image acquisition because of using only one type of visible-light banknote image.	Currency types need to be manually selected.
	Using Multiple Sensors	- Using fuzzy system on VR and NIRT banknote images [14]. - Using CNN with VR and IRT images for multinational banknote fitness classification [11].	Performance can be enhanced by using multiple imaging sensors.	Complexity and expensiveness in the implementation of hardware.
Banknote type and fitness classification (proposed method)		Using CNN for banknote recognition and fitness classification of banknotes from multiple countries with VR and IRT images.	Take advantage of deep learning technique on CNN for a large number of classes when combining banknote recognition and fitness classification into one classifier.	Time consuming procedure for CNN training is required.

**Table 2:** Summary of banknote datasets used for experiments in the previous studies (Ref: Reference(s), N/A: Not Available).

Category		Ref.	Currency Type	Output Description	Dataset Availability
Banknote Recognition	Single Currency Recognition	[3]	INR, AUD, EUR, SAR, USD	2 denominations for each of INR, AUD, EUR, and SAR. USD was not reported.	N/A
		[4]	USD, RMB, EUR	24 classes of USD, 20 classes of RMB, and 28 classes of EUR.	N/A
		[5]	USD, Angola (AOA), Malawi (MWK), South Africa (ZAR)	68 classes of USD, 36 classes of AOA, 24 classes of MWK, and 40 classes of ZAR.	N/A
		[6]	Hongkong (HKD), Kazakhstan (KZT), Colombia (COP), USD	128 classes of HKD, 60 classes of KZT, 32 classes of COP, and 68 classes of USD.	N/A
	Multiple Currency Recognition	[7]	Japan (JPN), Italy (ITL), Spain (ESP), France (FRF)	23 denominations.	N/A
		[8]	KRW, USD, EUR, CNY, RUB	55 denominations.	N/A
		[9]	CNY, EUR, JPY, KRW, RUB, USD	248 classes of 62 denominations.	DMC-DB1 [9]
		[10]	23 countries (USD, RUB, KZT, JPY, INR, EUR, CNY, etc.)	101 denominations.	N/A
Banknote Fitness Classification		[11]	INR, KRW, USD	5 classes with 3 classes of case 1 (fit, normal and unfit) and 2 classes of case 2 (fit and unfit).	DF-DB2 [11]
		[12]	EUR, RUB	2 classes (fit and unfit).	N/A
		[14]	USD, KRW, INR	2 classes (fit and unfit).	N/A
		[15]	KRW, INR, USD	3 classes of KRW and INR (fit, normal, and unfit), 2 classes of USD (fit and unfit).	DF-DB1 [15]
Banknote type and fitness classification (proposed method)			INR, KRW, USD	116 classes of banknote kinds and fitness levels.	DF-DB3 [20]

**Table 3:** Architecture of AlexNet used in this study (unit: pixel).

Layer Name		Filter Size	Stride	Padding	Number of Filters	Output Feature Map Size
Image Input Layer						120 × 240 × 3
Conv1	Conv.	7 × 7 × 3	2	0	96	57 × 117 × 96
	CCN					
	Max Pooling	3 × 3	2	0		28 × 58 × 96
Conv2	Conv.	5 × 5 × 96	1	2	128	28 × 58 × 128
	CCN					
	Max Pooling	3 × 3	2	0		13 × 28 × 128
Conv3	Conv.	3 × 3 × 128	1	1	256	13 × 28 × 256
Conv4	Conv.	3 × 3 × 256	1	1	256	13 × 28 × 256
Conv5	Conv.	3 × 3 × 256	1	1	128	13 × 28 × 128
	Max Pooling	3 × 3	2	0		6 × 13 × 128
Fully Connected Layers	Fc1					4096
	Fc2					2048
	Dropout					
	Fc3					116
	Softmax					

**Table 4:** Architecture of GoogleNet used in this study. “Conv. 1×1 (a)” and “Conv. 1×1 (b)” denote the 1 × 1 convolutional layers used for 3 × 3 and 5 × 5 convolutional computing reduction, respectively, “Conv. 1×1 (c)” denotes the 1 × 1 convolutional layers used for 3 × 3 pooling dimensional matching (unit of filter size, stride, and feature map size: pixel).

Layer Name		Filter Size/Stride	Number of Filters							Output Feature Map Size
				Conv. 1 × 1	Conv. 1 × 1 (a)	Conv. 3 × 3	Conv. 1 × 1 (b)	Conv. 5 × 5	Conv. 1 × 1 (c)	
Image Input Layer										120 × 240 × 3
Conv1	Conv.	7 × 7/2	64							60 × 120 × 64
	Max Pooling	3 × 3/2								30 × 60 × 64
	CCN									
Conv2	Conv.	1 × 1/1	64							30 × 60 × 64
	Conv.	3 × 3/1	192							30 × 60 × 192
	CCN									
	Max Pooling	3 × 3/2								15 × 30 × 192
Conv3	Inception3a			64	96	128	16	32	32	15 × 30 × 256
	Inception3b			128	128	192	32	96	64	15 × 30 × 480
	Max Pooling	3 × 3/2								7 × 15 × 480
Conv4	Inception4a			192	96	208	16	48	64	7 × 15 × 512
	Inception4b			160	112	224	24	64	64	7 × 15 × 512
	Inception4c			128	128	256	24	64	64	7 × 15 × 512
	Inception4d			112	144	288	32	64	64	7 × 15 × 528
	Inception4e			256	160	320	32	128	128	7 × 15 × 832
	Max Pooling	3 × 3/2								3 × 7 × 832
Conv5	Inception5a			256	160	320	32	128	128	3 × 7 × 832
	Inception5b			384	192	384	48	128	128	3 × 7 × 1024
	Average Pooling	3 × 7/1								1 × 1 × 1024
Fully-Connected Layer	Dropout									
	Fc									116
	Softmax									

**Table 5:** Architecture of ResNet-18 used in this study (unit: pixels).

Layer Name			Filter Size	Stride	Padding	Number of Filters	Output Feature Map Size
Image Input Layer							120 × 240 × 3
Conv1	Conv.		7 × 7 × 3	2	3	64	60 × 120 × 64
	BN						
	Max Pooling		3 × 3	2	1		30 × 60 × 64
Conv2	Res2a	Conv.	3 × 3 × 64	1	1	64	30 × 60 × 64
		Conv.	3 × 3 × 64	1	1	64	
	Res2b	Conv.	3 × 3 × 64	1	1	64	30 × 60 × 64
		Conv.	3 × 3 × 64	1	1	64	
Conv3	Res3a	Conv.	3 × 3 × 64	2	1	128	15 × 30 × 128
		Conv.	3 × 3 × 128	1	1	128	
		Conv. (Shortcut)	1 × 1 × 64	2	0	128	
	Res3b	Conv.	3 × 3 × 128	1	1	128	15 × 30 × 128
		Conv.	3 × 3 × 128	1	1	128	
Conv4	Res4a	Conv.	3 × 3 × 128	2	1	256	8 × 15 × 256
		Conv.	3 × 3 × 256	1	1	256	
		Conv. (Shortcut)	1 × 1 × 128	2	0	256	
	Res4b	Conv.	3 × 3 × 256	1	1	256	8 × 15 × 256
		Conv.	3 × 3 × 256	1	1	256	
Conv5	Res5a	Conv.	3 × 3 × 256	2	1	512	4 × 8 × 512
		Conv.	3 × 3 × 512	1	1	512	
		Conv. (Shortcut)	1 × 1 × 256	2	0	512	
	Res5b	Conv.	3 × 3 × 512	1	1	512	4 × 8 × 512
		Conv.	3 × 3 × 512	1	1	512	
	Average Pooling		4 × 8		0		1 × 1 × 512
Fully-Connected Layers	Fc						116
	Softmax						

**Table 6:** Architecture of ResNet-50 used in this study (unit: pixels).

Layer Name			Filter Size	Stride	Padding	Number of Filters	Output Feature Map Size
Image Input Layer							120 × 240 × 3
Conv1	Conv.		7 × 7 × 3	2	3	64	60 × 120 × 64
	BN						
	Max Pooling		3 × 3	2	1		29 × 59 × 64
Conv2	Res2a	Conv.	1 × 1 × 64	1	0	64	29 × 59 × 256
		Conv.	3 × 3 × 64	1	1	64	
		Conv.	1 × 1 × 64	1	0	256	
		Conv. (Shortcut)	1 × 1 × 64	1	0	256	
	Res2b-c	Conv.	1 × 1 × 256	1	0	64	29 × 59 × 256
		Conv.	3 × 3 × 64	1	1	64	
		Conv.	1 × 1 × 64	1	0	256	
Conv3	Res3a	Conv.	1 × 1 × 256	2	0	128	15 × 30 × 512
		Conv.	3 × 3 × 128	1	1	128	
		Conv.	1 × 1 × 128	1	0	512	
		Conv. (Shortcut)	1 × 1 × 256	2	0	512	
	Res3b-d	Conv.	1 × 1 × 512	1	0	128	15 × 30 × 512
		Conv.	3 × 3 × 128	1	1	128	
		Conv.	1 × 1 × 128	1	0	512	
Conv4	Res4a	Conv.	1 × 1 × 512	2	0	256	8 × 15 × 1024
		Conv.	3 × 3 × 256	1	1	256	
		Conv.	1 × 1 × 256	1	0	1024	
		Conv. (Shortcut)	1 × 1 × 512	2	0	1024	
	Res4b-f	Conv.	1 × 1 × 1024	1	0	256	8 × 15 × 1024
		Conv.	3 × 3 × 256	1	1	256	
		Conv.	1 × 1 × 256	1	0	1024	
Conv5	Res5a	Conv.	1 × 1 × 1024	2	0	512	4 × 8 × 2048
		Conv.	3 × 3 × 512	1	1	512	
		Conv.	1 × 1 × 512	1	0	2048	
		Conv. (Shortcut)	1 × 1 × 1024	2	0	2048	
	Res5b-c	Conv.	1 × 1 × 2048	1	0	512	4 × 8 × 2048
		Conv.	3 × 3 × 512	1	1	512	
		Conv.	1 × 1 × 512	1	0	2048	
	Average Pooling		4 × 8	1	0		1 × 1 × 2048
Fully Connected Layers	Fc						116
	Softmax						

**Table 7:** Number of input banknotes in the experimental multinational banknote fitness dataset.

Banknote Type	Number of Banknotes			Number of Banknotes after Data Augmentation		
	Fit	Normal	Unfit	Fit	Normal	Unfit
INR10	1299	553	196	2598	2212	1960
INR20	898	456	57	2694	2280	1425
INR50	719	235	206	1438	1175	2060
INR100	1477	1464	243	2954	2928	1944
INR500	1399	435	130	2798	2175	1950
INR1000	153	755	71	1530	2265	1775
KRW1000	3690	3344	2695	3690	3344	2695
KRW5000	3861	3291	3196	3861	4045	3196
KRW10000	3900	3779	N/A	3900	3779	N/A
KRW50000	3794	3799	N/A	3794	3799	N/A
USD5	177	N/A	111	3540	N/A	2775
USD10	384	N/A	83	3072	N/A	2075
USD20	390	N/A	51	3120	N/A	1275
USD50	851	N/A	42	4255	N/A	1050
USD100	772	N/A	90	3860	N/A	2250

**Table 8:** Comparative experimental results of the proposed method between with and without banknote region segmentation.

Method	AlexNet			ResNet-18		
	Banknote Recognition Accuracy	Fitness Classification Accuracy	Overall Accuracy	Banknote Recognition Accuracy	Fitness Classification Accuracy	Overall Accuracy
Using Original Banknote Image	99.535	97.035	96.773	99.608	97.532	97.408
Using Segmented Banknote Image (Proposed Method)	99.935	97.928	97.926	99.936	97.678	97.690

**Table 9:** Comparison of banknote type and fitness classification and banknote fitness value estimation results by our proposed method to that of previous method. “RMSE” denotes root-mean-squared error, “AQE” denotes average quantization error.

Method		Banknote Type and Fitness Classification Accuracy (unit: %)			Banknote Fitness Value Estimation	
		Banknote Recognition Accuracy	Fitness Classification Accuracy	Overall Accuracy	RMSE	AQE
Using VR Images and AlexNet [9,15]		99.955	95.040	95.038	1.048	0.0688
Using IRT, VR images and CNNs (Proposed Method)	AlexNet	99.935	97.928	97.926	0.920	0.0513
	ResNet-18	99.963	97.678	97.690	1.041	0.0522
